# Changes of Phenotypic Pattern in Functional Movement Disorders: A Prospective Cohort Study

**DOI:** 10.3389/fneur.2020.582215

**Published:** 2020-11-05

**Authors:** Aleksandra Tomić, Milica Ječmenica Lukić, Igor Petrović, Marina Svetel, Nataša Dragašević Mišković, Nikola Kresojević, Vladana Marković, Vladimir S. Kostić

**Affiliations:** Clinical Centre of Serbia, Faculty of Medicine, Clinic for Neurology, University of Belgrade, Belgrade, Serbia

**Keywords:** functional movement disorders, phenotypic progression, disease course, psychiatric disorders, psychosomatic disorders

## Abstract

**Introduction:** Functional movement disorders (FMD) refer to a group of movement disorders that present with clinical characteristics incongruent to those due to established pathophysiologic processes, as for example in the case of neurodegeneration or lesions. The aim of this study was to assess clinical features that contribute to the specific phenotypic presentations and disease course of FMD.

**Methods:** The study consisted of 100 patients with FMD treated at Clinic for Neurology, Clinical Center of Serbia, who were longitudinally observed. Comprehensive clinical and psychiatric assessment was performed at the baseline, when initial FMD phenotype was defined. Follow-up assessment of phenotypic pattern over the time and clinical course was done after 3.2 ± 2.5 years at average.

**Results:** We showed that 48% of FMD patients were prone to changes of phenotypic pattern during the disease course. Dystonia had tendency to remains as single and unchanged phenotype over the time (68.2%), while patients initially presented with Tremor, Gait disorder, Parkinsonism and Mixed phenotype were more susceptible to developing additional symptoms (62.5, 50, and 100%, respectively). Higher levels of somatoform experiences (*p* = 0.033, Exp(B) = 1.082) and higher motor severity (*p* = 0.040, Exp(B) = 1.082) at baseline assessment were associated with an increased likelihood of further enriching of FMD phenotype with additional functional symptoms. Also, these patients more frequently reported pain, and had higher scores on majority of applied psychiatric scales, together with more frequent presence of major depressive disorder.

**Conclusion:** Results from this prospective study suggested tendency for progression and enrichment of functional symptoms in FMD patients over time. Besides functional core symptoms, other key psychological and physical features (like pain or multiple somatisations) were quite relevant for chronicity and significant dysability of FMD patients.

## Introduction

Functional movement disorders (FMD) refer to a group of disorders that includes tremor, dystonia, myoclonus, parkinsonism, speech, and gait disturbances, and other movement disorders that are incongruent with established clinical patterns due to different pathophysiologic processes, as for example in the case of lesions (1). FMD belong to a larger entity called functional neurological disorders (FND), formerly known as psychogenic or conversion disorders, comprising neurological symptoms that cannot be explained by classical neurological disease (2).

The clinical features of FMD, including paroxysmal onset, waxing, and waning course, migration of symptoms to different body parts, together with possible symptom replacement (the resolution of one symptom can be followed by the appearance of another) (3), speak in favor of the unpredictable and often elusive nature of such disorders, leaving both patients and their physicians in the field of uncertainty. Furthermore, additional physical features (such as pain, fatigue, bladder, or bowel problems, etc), as well as psychiatric comorbidities, are usually associated with FND/FMD and can have significant impact on outcome (4).

The unpredictability of such disorders is further reflected through the highly variable prognosis of different FND (5). Limited data from heterogeneous and mostly retrospective studies reported persistent or worse state in more than a third of FND patients, with functional dystonia having the worst outcome (6, 7). A combination of wide variety of neurological and psychiatric symptoms are likely to contribute to disability and reduced quality of life in patients with FMD/FND (4), even being comparable to those of patients with Parkinson's disease (5).

However, little is known about the course of individual FMD subtypes and possible changes of phenotype over time. Therefore, we performed a longitudinal study, aimed to assess the phenotypic heterogeneity and disease course in patients with FMD, as well as to identify baseline prognostic factors which might be related to the changes of FMD pattern and the overall outcome.

## Patients and Methods

### Inclusion Criteria and Inform Consent

One hundred and eight in- and out-patients, who were referred to our Movement Disorder Department, Clinic for Neurology (University of Belgrade), between December 2011 and December 2019, fulfilled the criteria for diagnosis of FMD, in accordance with the Diagnostic and Statistical Manual of Mental Disorders 5th Edition criteria (DSM-5) (2). The clinical characteristic of 100 out of 108 patients were able to comply with longitudinal design of the study and were subsequently included in this study. Eight patients were lost at follow-up (7 patients with dystonia and 1 with mixed FMD phenotype) and their baseline characteristics (age, age at onset, sex, education, disease duration, as well as in the severity of illness measured through the PMDS), did not differ from those who were finally included in the study.

Briefly, diagnosis is based on the requirement that the neurological symptoms are associated with “clinical evidence of incompatibility between symptoms and recognized neurological or medical conditions” (8), by using clinical examination signs (9) that allow FMD to be positively differentiated from other disorders, rather than defined by the absence of another condition. In addition, all patients had normal brain imaging (CT or MRI scan). DaT-SPECT was normal in all patients with parkinsonism presentation. Genetic tests for mutations in the DYT1 and DYT6 gene were negative in dystonia patients, as well as mutations in DYT11 and Parkin gene in cases resembling dystonia-myoclonus phenotype and with late onset foot/hand dystonia, respectively.

Written informed consent was obtained from all patients and the study was approved by the Ethical Committee of the Faculty of Medicine, University of Belgrade.

### Conceptual Approach

Our investigation contained several steps:

#### Baseline Assessment and Definition of Initial FMD Phenotype

At the study entry (baseline), detailed demographic and clinical interviews were performed. The age at onset was defined as the age of the first appearance of symptom(s) attributable to FMD according to history, supported by an interview with the caregivers, and by medical charts. During the semi-structured interview, the data on precipitating events (physical and/or psychical trauma), mode of onset, disease evolution, and current and/or previous treatment were assessed as well.

Based on neurological examination, performed by two experienced movement disorder specialists (VSK, IP), the following “core” functional symptoms were noted for each of the 100 patients from our cohort (2):

1. FMD symptoms (abnormal movements), including dystonia, tremor, myoclonus, parkinsonism, gait abnormalities;
2. Other FND symptoms, including weakness/paralysis, sensory loss/anesthesia; special sensory symptoms (visual, auditory, olfactory), speech symptoms, swallowing symptoms; seizures [psychogenic non-epileptic seizures (PNES)], and autonomic symptoms (bladder and bowel problems).

According to the presence of “core” FMD symptoms, patients were assigned to following phenotypes, at initial examination: (1) Dystonia, (2) Tremor, (3) Gait disorder, (4) Parkinsonism, (5) Mixed phenotype (combination of any FMD symptoms, or combination of FMD with other FND symptoms).

All patients further underwent detailed motor and psychiatric assessment at baseline. Global motor severity and disability of FMD was assessed by Psychogenic Movement Disorders Rating Scale (PMDRS) (10). Global cognitive assessment was performed using the Mini-Mental State Examination (MMSE) (11). Psychiatric evaluation included the Hamilton Depression Rating Scale (HDRS) (12), Beck Depression Inventory (BDI-II) (13) the Hamilton Anxiety Rating Scale (HARS) (14), Patient Health Questionnaire (PHQ-9) (15) the Apathy Evaluation Scale (AES) (16), the Non-motor Symptoms Questionnaire (NMSQ) (17), Life-events Checklist (LEC) (18), the Somatoform Dissociation Questionnaire (SDQ-20) (19), and the Dissociative Experiences Scale II (DES-II) (20). Additionally, in order to established the existence of psychiatric diagnosis at baseline, all FMD patients underwent psychiatric interview and diagnosis was assigned based on DSM-5 criteria (2).

#### Follow-Up Assessment, Change of Phenotypic Pattern Over the Time and Clinical Course

All of 100 patients were regularly followed-up in 4- to 6-month intervals. At final follow-up examination, after 3.2 ± 2.5 years at average (range 1–8 years), the detailed neurological examination and semi-structured interview were performed by same experienced neurologists, to comprise evolution of symptoms and clinical course.

Based on the presence of “core” functional symptoms at final examination, the evolution of phenotype was categorized as follows: (1) Unchanged FMD, where the same symptoms were present on both initial and final examination; and (2) Changed, FMD plus phenotype (FMD plus), where patients developed additional FMD and/or FND symptoms at final examination, named as “Dystonia plus,” “Tremor plus,” “Parkinsonism plus,” “Gait disorder plus,” and “Mixed plus.”

Furthermore, to examine the clinical course of the disease, irrespective of the phenomenology changes on each follow-up visit, we scored patients as follows: (1) better or without symptoms; (2) unchanged; or (3) worse. Based on these criteria, at final examination disease course (final outcome) was defined. “Final outcome” was marked as: (1) progressive (continuous worsening of the same or other symptoms of FMD/FND, without periods of improvement); (2) fluctuating or relapse-remitting (RR) (with improvements or remissions of previous symptoms, but with subsequent relapses consisted of the same or other FMD or FND); (3) stationary (patients were without improvement or worsening of initial symptoms of FMD or FND, as well as with no additional symptoms).

### Statistical Analysis

The IBM SPSS 23.0 statistical software package was used in the statistical analysis.

The proportional distribution of categorical variables was analyzed with the chi-squared test. To identify differences between two and multiple groups means, *t*-test/Mann–Whitney U-test with Bonferroni correction (depending whether were data distributed normally or not) and ANOVA corrected for multiple testing (Games–Howell *post-hoc* test) were used, respectively.

A binominal logistic regression (forward stepwise variable selection method) was performed to ascertain the effects of baseline demographic, motor, and psychiatric variables on the likelihood that patient develop one or more FMD over the time (Unchanged FMD or FMD plus phenotype). After fulfilling all necessary assumptions for such analysis, following independent variables were entered in the model: gender, education, age at onset, disease duration, the presence of physical trauma preceding the onset, the presence of psychical trauma preceding the onset, PMDRS, LEC, HDRS, HARS, AES, NMSQ, SDQ 20, DES II).

Statistical significance was defined as a *p* < 0.05.

## Results

### Baseline Demographic and Clinical Characteristics of the FMD Cohort

Our study comprised 100 patients with FMD, with clear female predominance in gender distribution of our cohort (80 females; 80.0%). The mean age at onset was 44.5 ± 13.5 years, with disease duration of 6.1 ± 5.8 years. Acute, sudden onset of FMD symptoms was identified in 80 patients (80.0%). Psychological stressor (interpersonal conflicts, complex family, and partnership dynamics) preceding FMD onset was present in 66% of patients, while almost one quarter of patients (24%) reported physical trauma as precipitating event. More than two thirds of patients had pain in the body part affected with functional symptoms (67.0%), while half of them reported the presence of pain in unaffected region (Table 1).

**Table 1:** Demographic and clinical characteristics of 100 patients with functional movement disorders at initial examination.

**Variables**	
Female, *N* (%)	80 (80.0)
Education (years)	11.8 ± 2.0 [6–18]
Age (years)	50.7 ± 13.6 [19–75]
Age at onset (years)	44.5 ± 13.5 [13–72]
Disease duration (years)	6.1 ± 5.8 [1–28]
**Mode of onset**	
Gradual, *N* (%)	20 (20.0)
Sudden, *N* (%)	80 (80.0)
**Trauma preceding the disease onset**	
Physical trauma, *N* (%)	24 (24.0)
Psychical trauma, *N* (%)	65 (65.0)
**Presence of pain**	
Affected body part, *N* (%)	67 (67.0)
Unaffected body part, *N* (%)	50 (50.0)
**Global cognitive assessment**	
MMSE	28.1 ± 1.7 [25–30]
**Motor status**	
PMDRS total phenomenology score	11.0 ± 5.0 [0–33]
PMDRS total functional score	6.6 ± 4.0 [0–12]
PMDRS total score	17.7 ± 6.9 [4–42]
**Psychiatric tretament**	
Prior psychiatric treatment, *N* (%)	50 (50.0)
Current psychiatric treatment, *N* (%)	71 (71.0)
**Psychiatric scales**	
HDRS	15.8 ± 9.6 [0–42]
BDI	16.8 ± 11.6 [0–54]
HARS	15.0 ± 10.9 [0–46]
PHQ-9	10.5 ± 7.3 [0–24]
AES	17.6 ± 10.3 [0–42]
DES-II	3.3 ± 6.2 [0–25]
SDQ 20	27.8 ± 9.5 [20–58]
NMSQ	9.1 ± 5.6 [0–24]
LEC	2.4 ± 2.1 [0–12]
**Diagnosis according to DSM-5 criteria**	
MDD single episode, *N* (%)	23 (23.0)
Dysthimia, *N* (%)	10 (10.0)
MDD reccurent, *N* (%)	22 (22.0)
MDD psychotic, *N* (%)	1 (1.0)
Bipolar affective disorder, *N* (%)	1 (1.0)
Specific phobia, *N* (%)	2 (2.0)
Panic attacks, *N* (%)	1 (1.0)
Generalized anxiety disorder, *N* (%)	1 (1.0)
Anxious-depressive disorder, *N* (%)	4 (4.0)
Sedative-related disorder, *N* (%)	1 (1.0)
Adjustment disorder, *N* (%)	2 (2.0)
Somatisation, *N* (%)	4 (4.0)
Schizophrenia, *N* (%)	2 (2.0)
Delusional disorder, *N* (%)	1 (1.0)
Borderline personality disorder, *N* (%)	3 (3.0)
Undifferentiated personality disorder, *N* (%)	5 (5.0)

*Values are presented as means ± standard deviations [range], unless noted otherwise. MMSE, Mini-Mental State Examination; PMDRS, Psychogenic Movement Disorders Rating Scale; HDRS, Hamilton Depression Rating Scale; BDI, Beck Depression Inventory; HARS, Hamilton Anxiety Rating Scale; PHQ-9, Patient Health Questionnaire-9; AES, Apathy scale; DES-II, Dissociative Experience Scale II; SDQ-20, Somatoform Disorders Questionnaire; NMSQ, Non-Motor Symptom Questionnaire; ns, non-significant; LEC, Life events checklist; MDD, major depressive disorder*.

The baseline motor and psychiatric characteristics of our cohort are presented in Table 1. Half of patients underwent psychiatric treatment prior to the diagnosis of FMD, while 71% of patients were under psychiatric treatment at baseline examination (Table 1). According to DSM-5 criteria, the most commonly established psychiatric diagnoses were major depressive disorder (MDD), both in the form of single (23%) or recurrent episodes (22%), followed by dysthymia diagnosed in 10% of patients.

### The Prevalence of Functional Neurological Symptoms at First and Final Examination

**Figure 1:**
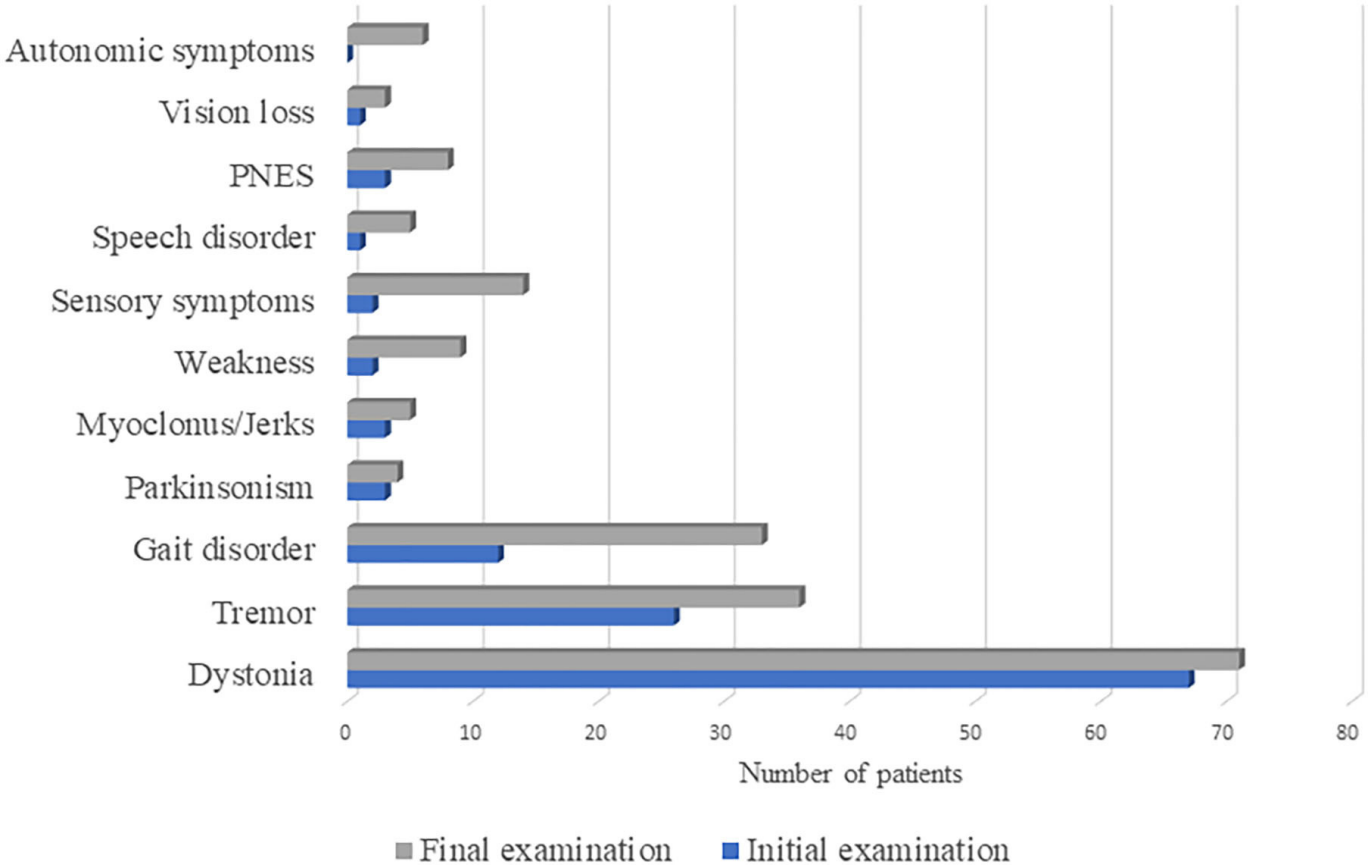
The prevalence of functional neurological symptoms at first and final examination in 100 patients diagnosed with functional movement disorder. PNES, psychogenic non-epileptic seizures.

The prevalence of functional neurological symptoms at first and final examination is presented in Figure 1. The most prevalent symptom was dystonia, initially present in 67 and lastly in 71 patients, followed by tremor, with rising prevalence from 26 to 36 percentages over time. Gait disorder was observed in only 12% at initial examination, while at the final examination one third of patients developed such symptoms. Parkinsonism and myoclonus/jerks were observed in <5% of patients, both at initial and final examination. Other functional neurological symptoms were observed only in the presence of FMD symptoms, which prevalence at initial and final examination were as follows: weakness (palsy) in 2 and 9 patients, sensory loss in 2 and 14 patients, speech disorder in 1 and 5 patients, PNES in 3 and 8 patients, vision loss in 1 and 3 patients, respectively. Autonomic symptoms were only present at final examination and reported by 6 patients.

### The Evolution of FMD Phenotypes and Disease Course Over the Follow-Up Period

At study entry, there was a predominance of Dystonia phenotype (63%), followed by Tremor in 16%, Gait disorder in 6%, and Parkinsonism in 3%, while Mixed phenotype was identified in 12% of investigated cohort. Myoclonus/jerks occurred only in the presence of other FMD symptoms, therefore hiding under the group of Mixed phenotype. We did not find significant differences between these groups in any baseline demographic and clinical characteristics, except that patients with parkinsonism have shown significantly higher score on PHQ-9, in contrast to dystonia, tremor and mixed baseline FMD phenotype (*p* < 0.05, ANOVA, *post hoc* Games Hallms).

**Figure 2:**
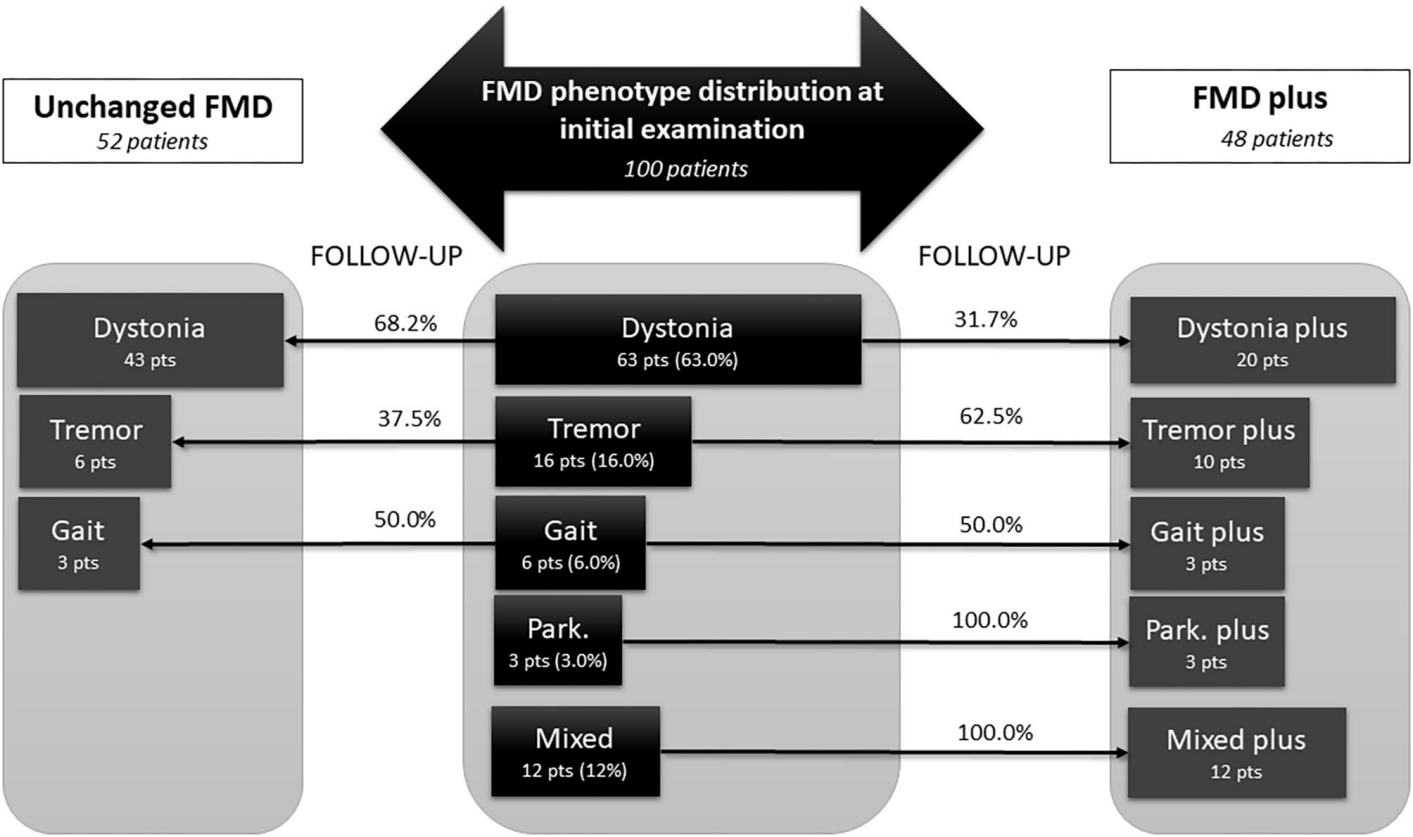
Changes in phenotypic pattern of functional movement disorder from baseline to final examination, after mean follow up of 3.2 ± 2.5 years. Central panel represent phenotype distribution at initial examination in 100 patients diagnosed with FMD, either as a single phenotype (dystonia, tremor, gait disorder, or parkinsonism) or as a mixed phenotype, including combination of any FMD symptoms, or combination of FMD with other FND symptoms (like palsy, speech disorder, sensory loss, vision loss, PNES, etc). Lateral parts depict patients at final examination, after mean follow-up of 3.2 ± 2.5 years. Fifty-two patients that remained unchanged, with single FMD phenotype (dystonia, tremor, gait disorder) are present in the left panel. The right panel, represent the FMD phenotypes that have been changed and become FMD plus phenotype at the final examination, i.e., besides initial FMD phenotype, these patients developed additional FMD or FND symptoms (for instance: dystonia plus). All patients with Mixed phenotype at initial examination become Mixed plus because they developed additional FMD or FND symptoms at final examination. FMD, functional movement disorders; FND, functional neurological disorders; Park, parkinsonism; pts, patients.

The classification of phenotype at final examination was performed according to criteria defined in methodology. The distribution of FMD phenotypes at initial and final examination is presented at Figure 2. After follow-up period of 3.2 ± 2.5 years, 48% of patients enriched clinical presentation with additional functional neurological symptoms (FMD plus), while 52% of patients remained unchanged (Unchanged FMD). At final examination, dystonia predominantly remained in the form of single, unchanged FMD phenotype (68.2%), while 31.7% progressed into Dystonia plus. Half of the patients with initial Gait disorder progressed into Gait plus phenotype. Almost two thirds of patients (62.5%) who initially presented with Tremor, eventually developed additional functional neurological symptoms over the follow up period, while all patients who initially had Parkinsonism and Mixed phenotype, have evolved into FMD plus.

**Figure 3:**
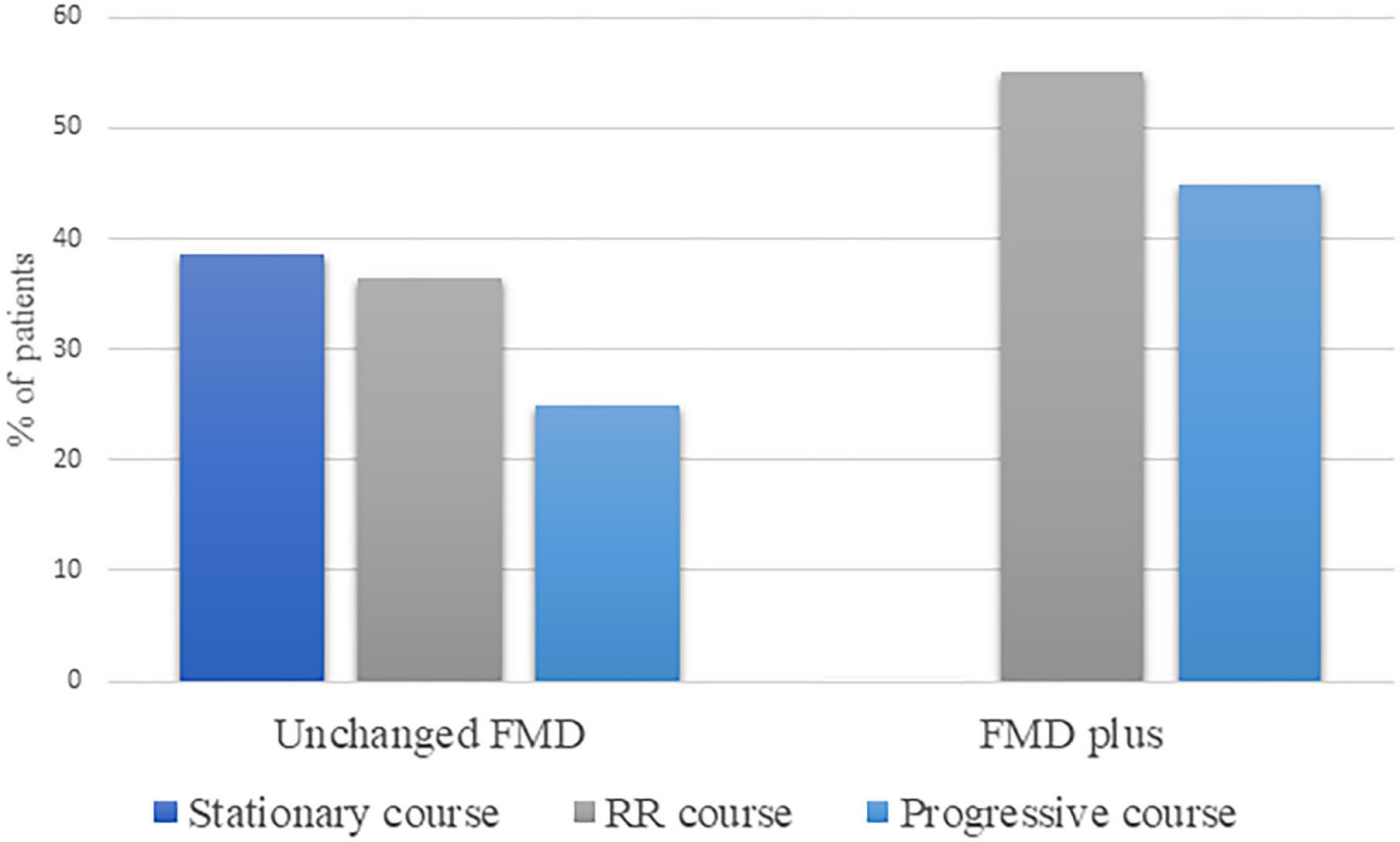
Disease course in terms of progression, within Unchanged FMD and FMD plus phenotype. RR, relapse-remitting; FMD, functional movement disorder.

We have also evaluated disease course in terms of progression of FMD and identified stationary disease course in 20%, RR in 46%, and progressive in 34% of patients, over the follow-up period. Patients with Unchanged FMD, exhibit stationary course in 38.5% of cases, while the majority of patients have shown changes in the course of the disease, either as fluctuating, RR (36.5%) or progressive course (25%) (Figure 3). On the other hand, all patients with FMD plus, have shown overall changes in the course of the disease, in the form of RR (55.1%) or progressive course (44.9%).

### Associations Between Baseline Characteristics and, Respectively, Unchanged FMD and FMD Plus Phenotype

Comparison of baseline demographic and clinical characteristics of FMD patients that eventually developed Unchanged FMD or FMD plus phenotypes, is presented in Table 2. Patients that evolved into FMD plus more frequently reported pain in unaffected body parts. Furthermore, these patients had more severe motor impairment (higher scores of PMDRS), as well as higher AES, PHQ-9, HAMA, DES-II, SDQ 20, NMSQ scores. In addition, patients who developed FMD plus relative to group of Unchanged FMD, were diagnosed more frequently with recurrent major depressive disorder at baseline, according to DMS-5 criteria (Table 2).

**Table 2:** Comparison of initial demographic and clinical characteristics of patients that developed Unchanged FMD or FMD plus phenotype at final examination.

**Variable at initial examination**	**Phenotype at final examination**		
	**Unchanged FMD phenotype** ***N* = 52**	**FMD plus phenotype** ***N* = 48**	***p*-value**
Female, *N* (%)	41 (78.8)	39 (81.2)	ns
Education (years)	11.9 ± 2.1 [8–18]	11.7 ± 1.9 [6–16]	ns
Age (years)	49.5 ± 15.1 [19–75]	52.0 ± 11.7 [22–74]	ns
Age at onset (years)	43.2 ± 14.9 [13–72]	46.0 ± 11.7 [14–70]	ns
Disease duration (years)	6.0 ± 5.8 [1–28]	6.2 ± 5.9 [1–26]	ns
**Mode of onset**			
Gradual, *N* (%)	11 (21.1)	9 (18.7)	ns
Sudden, *N* (%)	41 (78.8)	39 (81.2)	ns
**Trauma preceding the disease onset**			
Physical trauma, *N* (%)	11 (21.1)	13 (28.3)	ns
Psychical trauma, *N* (%)	30 (57.7)	34 (73.9)	ns
**Presence of pain**			
Affected body part, *N* (%)	34 (66.7)	33 (71.7)	ns
Unaffected body part, *N* (%)	20 (39.2)	30 (65.2)	0.009
**Global cognitive assessment**			
MMSE	28.3 ± 1.6 [25–30]	27.8 ± 1.7 [25–30]	ns
**Motor status**			
PMDRS total phenomenology score	9.9 ± 3.5 [0–18]	12.2 ± 6.1 [0–33]	0.020
PMDRS total functional score	5.7 ± 4.6 [0–12]	7.6 ± 3.0 [0–12]	0.018
PMDRS total score	15.8 ± 6.3 [4–30]	19.9 ± 7.0 [7–42]	0.003
**Psychiatric treatment**			
Prior psychiatric treatment, *N* (%)	27 (55.1)	23 (51.1)	ns
Current psychiatric treatment, *N* (%)	34 (69.4)	37 (82.2)	ns
**Psyhiatric scales**			
HDRS	14.0 ± 9.3 [0–36]	17.7 ± 9.7 [2–42]	ns
BDI	15.2 ± 10.6 [0–47]	18.5 ± 12.4 [0–54]	ns
HARS	12.4 ± 8.1 [0–31]	17.9 ± 12.8 [1–46]	0.018
PHQ-9	9.0 ± 6.5 [0–22]	12.1 ± 7.7 [1–24]	0.033
AES	14.7 ± 8.7 [0–36]	20.8 ± 11.0 [0–42]	0.003
DES-II	1.5 ± 4.1 [0–25]	5.2 ± 7.7 [0–24]	0.003
SDQ 20	25.2 ± 7.1 [20–50]	30.7 ± 11.0 [20–58]	0.004
NMSQ	7.3 ± 5.0 [0–24]	11.0 ± 5.7 [2–22]	0.001
LEC	2.3 ± 2.3 [0.12]	2.6 ± 1.9 [0–7]	ns
**Diagnosis according to DSM-5 criteria**			
MDD single episode, *N* (%)	12 (23.0)	11 (22.9)	ns
Dysthimia, *N* (%)	6 (11.5)	4 (8.3)	ns
MDD reccurent, *N* (%)	7 (13.5)	15 (31.2)	0.034
MDD psychotic, *N* (%)	1 (1.9)	0 (0)	ns
Bipolar affective disorder, *N* (%)	1 (1.9)	0 (0)	ns
Specific phobia, *N* (%)	2 (3.8)	0 (0)	ns
Panic attacks, *N* (%)	0 (0)	1 (2.1)	ns
Generalized anxiety disorder, *N* (%)	1 (1.9)	0 (0)	ns
Anxious-depressive disorder, *N* (%)	3 (5.8)	1 (2.1)	ns
Sedative-related disorder, *N* (%)	1 (1.9)	0 (0)	ns
Adjustment disorder, *N* (%)	1 (1.9)	1 (2.1)	ns
Somatisation, *N* (%)	1 (1.9)	3 (6.2)	ns
Schizophrenia, *N* (%)	2 (3.8)	0 (0)	ns
Delusional disorder, *N* (%)	1 (1.9)	0 (0)	ns
Borderline personality disorder, *N* (%)	1 (1.9)	2 (4.2)	ns
Undifferentiated personality disorder, *N* (%)	2 (3.8)	3 (6.2)	ns

*Values are presented as means ± standard deviations [range], unless noted otherwise. MMSE, Mini-Mental State Examination; PMDRS, Psychogenic Movement Disorders Rating Scale; HDRS, Hamilton Depression Rating Scale; BDI, Beck Depression Inventory; HARS, Hamilton Anxiety Rating Scale; PHQ-9, Patient Health Questionnaire-9; AES, Apathy scale; DES-II, Dissociative Experience Scale II; SDQ-20, Somatoform Disorders Questionnaire; NMSQ, Non-Motor Symptom Questionnaire; ns, non-significant; LEC, Life events checklist; MDD, major depressive disorder*.

In order to examine the effects of baseline demographic, motor, and psychiatric variables on the likelihood that patient develop one or more additional FND over the time, the binary logistic regression model was performed. The model was statistically significant (χ^2^ = 19.626, *p* < 0.001), explained 25.6% (Nagelkerke *R*^2^) of the variance in phenotype and correctly classified 71.7% of cases. Increasing in SDQ-20 and PMDRD scores were associated with an increased likelihood of exhibiting FMD plus phenotype (Table 3).

**Table 3:** Associations between baseline characteristics and respectively unchanged FMD and FMD plus phenotype (binominal logistic regression).

**Model summary**	**Chi^2^ = 19.626; *p* < 0.001; Cox & Snell *R*^2^ = 0.192;** **Nagelkerke *R*^2^ = 0.256, Correct classification 71.7%**					
**Parameters of regression**	**B**	**S.E**.	**Wald**	***p***	**Exp(B)**	**95% C.I. for Exp(B)**
PMDRS total	0.079	0.038	4.234	0.040	1.082	1.004–1.166
SDQ-20	0.055	0.026	4.523	0.033	1.057	1.004–1.112

*The significant results (p < 0.05), from binomial logistic regression models. A logistic regression was performed to ascertain the effects of different demographic, clinical, and psychiatric variables, on the likelihood that patient develop one or more functional movement disorder over the time (Unchanged FMD or FMD plus phenotype)*.

## Discussion

The main finding of our prospective study of 100 FMD patients is that 48% of patients are prone to changes of phenotypic pattern during the disease course. Patients initially presented with Tremor, Gait disorder, Parkinsonism, and Mixed phenotype are more susceptible to developing additional symptoms, while dystonia has tendency to remains as single and unchanged phenotype over the time. Higher levels of somatoform experiences and higher motor severity, expressed through the higher scores on SDQ-20 and PMDRD scales at baseline assessment, were associated with an increased likelihood of further enriching of FMD phenotype with additional functional symptoms.

Various studies reported that the most common presentations of FMD were tremor (40.6–50% of FMD patients) and dystonia (17.2–18%), with parkinsonism, tics, myoclonus and chorea being less common (3, 21–24). Our results confirmed functional dystonia and tremor as the most frequent symptoms, both initially (67 and 26%, respectively), and at final examination (71 and 36% patients, respectively). The inverse order observed in our study (dystonia more frequent than tremor in our cohort) in contrast to previous findings, was probably due to the primary research orientation of our Department toward dystonic disorders. Functional gait disorder was the third most common symptom in our sample. It was present in 12% of patients initially, while after follow-up it was almost 3 times more frequent. However, gait disorder was predominantly combined with other FMD/FND. As isolated initial FMD phenotype it was reported in only 6 patients, with half of them evolving to Gait disorder plus after developing additional FMD/FND symptoms. Thus, we confirmed that functional gait disturbances usually take part in more complex FMD/FND clinical presentations (25). Since functional gait manifestations were often difficult to classify (26), we suggested that the presence of other positively identifiable functional symptoms in the same patient might be a useful, although not certain, diagnostic clue.

As expected, parkinsonism was rare in our sample, initially, and during natural course of disease (3 and 4% patients, respectively). Functional parkinsonism might be often associated with underlying Parkinson's disease (27), and also almost a third of patients with functional parkinsonism had a family history of tremor or parkinsonism (28). All our patients with isolated parkinsonian phenotype at initial examination progressed to “parkinsonism plus” phenotype, which made us more certain that we are dealing with functional deficit, together with normal findings on DaT-SPECT. In addition, the only differences in baseline clinical assessment between different FMD phenotypes, were found in PHQ-9 scale, where patients

with parkinsonism phenotype have reported more depressive symptoms in contrast to other FMD phenotypes. Previous study has shown that 56% of patients with functional parkinsonism had a psychiatric disorder, mostly depression (28), suggesting the complex interplay between these symptoms. On the other hand, depressive symptoms related to psychomotor retardation might mimic bradykinesia (1).

Even though paroxysmal jerks or myoclonus were presented as additional movements combined with more prominent dystonia or tremor, both initially and through time, recently described phenotype of paroxysmal movement disorders (29) was not identified in our cohort.

During the follow-up period, we found almost equal proportion of patients who were prone to phenotypic changes and those with preserved phenotype. Patients initially presenting with Tremor, Gait disorder, Parkinsonism, and Mixed phenotype are most susceptible to developing plus symptoms, while patients with Dystonia phenotype predominantly has tendency to remain as single FMD symptom over the time. This is specifically significant for patients with fixed dystonia who are characterized by severe and spreading isolated dystonia in majority of cases, as we showed in our previous study (30).

It is important to emphasize here, that stable and unchanged phenotype, does not necessary imply stationary disease course and favorable prognosis. Remarkably, majority of patients which were not prone to phenotypic changes, presented with RR and progressive disease course (for example, patients with isolated focal dystonia that progresses to segmental or hemidystonia). Therefore, phenomenology and phenotype of involuntary movements are important, but not the only factor that determines the severity of clinical presentations. In other words, the severity and progression of the disease should not be equated with the appearance of new functional symptoms.

One more interesting point is that none of our patients had complete change in phenotype, despite development of numerous new symptoms over the time.

Another question that we addressed in our study refer to identification of possible cluemarks from initial clinical examination, which could help us in further prognosis of FMD. Indeed, patients that are prone to develop additional functional symptoms during disease course, have specific sensory, motor, and psychological characteristics at disease onset, in contrast to unchanged FMD group. Initially, these patients more frequently reported pain in unaffected body parts, presence of psychosomatic disorders, had more severe motor impairment and higher scores on majority of applied psychiatric scales. These data indicate that motor symptoms, along with psychiatric and non-motor symptoms, are initially far more severe in patients who tend to develop multiple functional features over time.

Previously, it was shown that patients with FMD in general, had a high frequency of lifetime anxiety disorders (61.9%), major depression (42.9%) and personality disorders (45%) (31). However, more recent evidence suggested that these patients rather scored high in depression and anxiety in validated clinical scale than they had diagnosis of psychiatric or personality disorders (32, 33). In line with this, we should stress here that our FMD plus patients with complex phenotype, had not just higher depressive, anxiety and apathy scores on clinical scales, but were also diagnosed more frequently with MDD and dysthymia, according to DSM-5 diagnostic criteria, in contrast to patients with Unchanged FMD.

However, the only independent predictors of phenotypic changes over the disease course, are related to the presence of somatoform experience and higher motor severity, at baseline. Expression of emotions primarily through the somatic channel in the form of somatic and neurological symptoms due to deficient processing (mentalization) is the psychological basis of conversion disorders and disorders characterized by somatization (34). The recognition of somatoform dissociative symptoms in patients with FMD might be very important as it should alert clinicians for possible progression of functional symptoms over the time. In addition, pathologic fragmentation of bodily (somatoform) experiences (19) in FMD was associated with worse outcome (6). Besides psychological factors, increasing PMDRS score was a predictor of having FMD plus phenotype, suggesting that more complex initial motor presentation might be a red fleg for further evolution of more severe functional symptoms.

These results support the concept of the complex and bidirectional interplay between psychological background and progression of motor symptoms. Also, different physical features, like pain or multiple somatisations make a significant impact to disease presentation and evolution. In other words, besides functional core symptoms, other key psychological and physical features are of quite relevance for chronicity and significant dysability of FMD/FND patients (4).

One of the limitations of the study is an unbalanced sample, with predominance of dystonia, explained by recruitment bias of previous research. Still, dystonia and tremor are the most prevalent FMD in our cohort, and the distribution of other FMD, reflects the proportion seen in other studies. Another limitation is related to the lack of systematized treatment information that patients received during the follow-up period, which could affect the outcome. Treatment was not limited to one institution, patients received individualized treatments indicated by physicians of different specialties, often with poor compliance to the suggested treatment options. In the absence of structured and uniform tretament protocol, it can be argued that this cohort would rather reflect a study of the natural course of the disease, than be influenced by treatment bias. Taking into account the evolving recommendations for FND care, as well as differences in the health care system, more research is needed to comprehensively investigate the link between disease outcome and “standardized treatments.” The main strength of this study is primarily its prospective nature, together with a long follow-up period, large sample size, mixed cohort, and the use of a wide range of motor and psychometric scales, making us more confident in interpreting these results, despite obvious limitations.

In conclusion, results from our prospective study suggest that significant number of FMD patients are prone to phenotypic changes over time. We believe that this fluidity of phenotype further supports the recognized diagnostic criteria of inconsistency and incongruence. The greater severity of motor symptoms at the onset of the disease is not the only predictor of phenotypic changes in FMD over the time, but attention must be focused on subtle signs of somatoform experiences. These data imply and argue a careful and comprehensive approach, as well as early recognition and targeted therapeutic approach in FMD.

## Data Availability Statement

The raw data supporting the conclusions of this article will be made available by the authors, without undue reservation.

## Ethics Statement

The studies involving human participants were reviewed and approved by Ethical Committee of the Faculty of Medicine, University of Belgrade. The patients/participants provided their written informed consent to participate in this study.

## Author Contributions

AT, IP, VK, and MJ contributed to conception and design of the study. AT wrote the first draft of the manuscript. MJ performed the statistical analysis. ND, MS, NK, and VM contributed to organization and data collection. All authors contributed to manuscript revision, read, and approved the submitted version.

## Conflict of Interest

IP has received speaker honoraria from Actavis and Salveo. VK has received speaker honoraria from Roche and Alkaloid; and receives research supports from the Swiss Pharm and Serbian Ministry of Education, Science, and Development and Serbian Academy of Sciences and Art. The remaining authors declare that the research was conducted in the absence of any commercial or financial relationships that could be construed as a potential conflict of interest.
